# Alzheimer’s Disease: Models and Molecular Mechanisms Informing Disease and Treatments

**DOI:** 10.3390/bioengineering11010045

**Published:** 2024-01-01

**Authors:** Kaden L. Nystuen, Shannon M. McNamee, Monica Akula, Kristina M. Holton, Margaret M. DeAngelis, Neena B. Haider

**Affiliations:** 1 Department of Chemical Engineering, University of Massachusetts Amherst, Amherst, MA 01003, USA; 2 Schepens Eye Research Institute, Massachusetts Eye and Ear, Department of Ophthalmology, Harvard Medical School, Boston, MA 02114, USA; 3 Department of Stem Cell and Regenerative Biology, Harvard University, Cambridge, MA 02138, USA; 4 Harvard Stem Cell Institute, Cambridge, MA 02138, USA; 5 Stanley Center for Psychiatric Research, Broad Institute of MIT and Harvard, Cambridge, MA 02142, USA; 6 Department of Ophthalmology, Jacobs School of Medicine and Biomedical Sciences, University at Buffalo, Buffalo, NY 14203, USA; 7 Department of Cell Biology, Harvard Medical School, Boston, MA 02115, USA

**Keywords:** Alzheimer’s Disease, amyloid β, Tau

## Abstract

Alzheimer’s Disease (AD) is a complex neurodegenerative disease resulting in progressive loss of memory, language and motor abilities caused by cortical and hippocampal degeneration. This review captures the landscape of understanding of AD pathology, diagnostics, and current therapies. Two major mechanisms direct AD pathology: (1) accumulation of amyloid β (Aβ) plaque and (2) tau-derived neurofibrillary tangles (NFT). The most common variants in the Aβ pathway in *APP*, *PSEN1,* and *PSEN2* are largely responsible for early-onset AD (EOAD), while *MAPT*, *APOE*, *TREM2* and *ABCA7* have a modifying effect on late-onset AD (LOAD). More recent studies implicate chaperone proteins and Aβ degrading proteins in AD. Several tests, such as cognitive function, brain imaging, and cerebral spinal fluid (CSF) and blood tests, are used for AD diagnosis. Additionally, several biomarkers seem to have a unique AD specific combination of expression and could potentially be used in improved, less invasive diagnostics. In addition to genetic perturbations, environmental influences, such as altered gut microbiome signatures, affect AD. Effective AD treatments have been challenging to develop. Currently, there are several FDA approved drugs (cholinesterase inhibitors, Aß-targeting antibodies and an NMDA antagonist) that could mitigate AD rate of decline and symptoms of distress.

## 1. Introduction

Dementia is a complex neurodegenerative disease characterized by a broad range of neurological symptoms and pathologies, and is among the leading causes of death worldwide. Several subtypes of dementia have been classified thus far to encompass the various phenotypes and pathologies. The most common subtypes include Alzheimer’s Disease (AD), Lewy body dementia (LBD), Parkinson’s disease dementia (PDD), frontotemporal dementia (FTD), prion-related dementia, and vascular dementia (VD) [1,2,3]. The defining characteristics of the non-vascular diseases are their underlying proteinopathies. AD is characterized by the accumulation of abnormal Aß and tau aggregates. Abnormal aggregates of α-synuclein, called Lewy bodies, are associated with LBD and PDD. FTD can have abnormal accumulation of aggregates of TAR DNA-binding protein 43 (TDP-43), fused in sarcoma (FUS), or tau, similar to AD. Finally, prion-related dementia is characterized by abnormal scrapie prion protein (PrP^Sc^) aggregates and sometimes Aß. Unlike other neurodegenerative conditions, vascular brain injury or stroke and not protein accumulation is associated with VD. Diagnosis of dementia subtypes is difficult to confirm due to the overlap sometimes present in clinical symptoms and is often not definitively confirmed until post-mortem autopsy [2]. This difficulty can lead to hesitation on the part of both patients and doctors in seeking a diagnosis and can result in a large number of misdiagnoses delaying proper treatment. Studies of adults in the US found that about 40% of older adults with probable dementia are underdiagnosed, and 53% with atypical presentations of EOAD are misdiagnosed [4,5]. 

AD is the leading cause of dementia and results in the progressive loss of memory, ability to converse, and motor function [6,7]. Women (approximately 20%) have a greater risk of AD compared to men (approximately 11%) with about two-thirds of all AD patients being female [8,9]. AD is divided into early-onset AD (EOAD) and late-onset AD (LOAD), which both have familial and sporadic occurrences. AD with familial autosomal dominant inheritance is rare, accounting for <1% of cases, and generally manifests in EOAD, whereas sporadic LOAD is the most common form [10]. Furthermore, early-onset AD accounts for 5–10% of all AD cases, and applies to people diagnosed between the ages of about 30–64, while late-onset AD includes people diagnosed at age 65 or older [11,12,13]. One of the greatest risk factors for AD is aging, and as science continues to extend the average human lifespan, the number of people affected by AD will continue to rise. AD is currently the seventh-leading cause of death in the United States with rates having increased by 145% [7,11]. EOAD may be less common; however, it still afflicts around 200,000 Americans between the ages of 30 and 64 [7,11,14]. LOAD affects about 1 in 9 people aged 65 and older, and 1 in 3 people 85 and older in the U.S. [7,15]. Currently, approximately 6.7 million Americans over 65 currently have AD, and 73% of them are 75 or older [7,15]. Worldwide, over 50 million people were living with AD in 2020, and this number is expected to increase to 152 million by the year 2050 as the population of people over 80 increases [6].

The progression of AD results in the destruction of neurons affecting all lobes of the brain, starting with the neurons associated with memory, language, and thought processing [7]. The manifestation time of observable symptoms of this neurodegeneration can vary greatly, sometimes appearing immediately, or up to 20 years later in severe cases [7]. This is likely to be attributed to the unique mutational load and environmental factors in each patient. People in the early/preclinical stages of disease are usually able to carry on everyday life with minimal assistance from others. However, as the neurodegeneration progresses, symptoms become more severe, and more assistance with basic tasks is required [6,16]. AD patients in the mild stages experience primarily changes in mood and personality along with some difficulty completing basic tasks [6]. As the disease progresses, moderate stage patients experience a loss of motor functions and cognitive decline with loss of the ability to remember, converse, and think [6,16]. As AD progresses further, severe neurodegeneration leads to further cognitive decline and patients are unable to remember family members’ names or make new memories, and become bedridden, with difficulty swallowing and urinating, eventually resulting in the patient’s death [6]. The variability in severity of disease progression and symptoms coupled with the short life expectancy following diagnosis, an average of 4–8 years in individuals with LOAD [7], necessitates development of earlier detection methods and more treatment alternatives than the simple symptom management currently used. Two new drug treatments were recently approved by the FDA, described later, to treat early and mild AD. However, there remains an unmet need for a treatment in moderate and severe patients.

### Diagnosis

**Figure 1:**
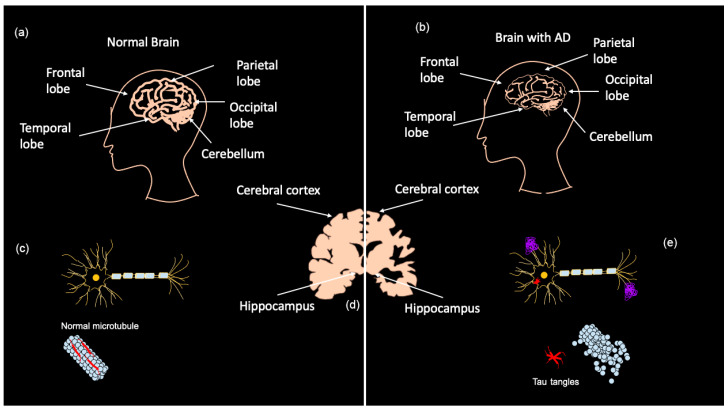
Brain matter degeneration in Alzheimer’s Disease (AD) patients. Normal brain matter (**a**) compared to brain matter in advanced AD patients (**b**). Brain regions affected are the hippocampus and the cortex (**d**), including the frontal, parietal, occipital and temporal lobes. Amyloid β (Aβ) plaque forms in the extracellular space of neurons in the AD brain, and tau-derived neurofibrillary tangles accumulate intracellularly (**e**) when compared with the healthy neuron (**c**). Adapted from Bagad, M. et al. (2013) [19].

Early diagnosis of AD is a major challenge as most symptoms do not often arise until the severe stages of neurodegeneration; however, the brain changes associated with those symptoms are believed to begin up to 20 years before clinical manifestation [7]. AD is currently diagnosed in several ways, ranging from memory tests and puzzles to brain scans and blood/CSF tests. Presently, two categories of biomarkers exist for AD: markers for amyloid plaque build-up in the brain and markers for neuronal injury [6]. Positron emission tomography (PET) shows amyloid and tau deposits in the brain, while cerebrospinal fluid (CSF) can show abnormal amyloid ß (Aß) and tau levels [6,7]. Tau CSF levels are also used as an indicator of neuronal injury along with magnetic resonance imaging (MRI) to detect brain atrophy [7]. As shown in Figure 1a–c, MRIs demonstrate that advanced AD patients have considerably less brain matter than healthy controls as a result of neurodegeneration. AD biomarkers detectable in CSF are Aß_1–42_, phosphorylated (P)-Tau, total (T)-Tau, and Neurogranin [17]. It should be noted that PET imaging and CSF testing are not frequently carried out. PET imaging is mostly only performed on patients participating in clinical trials, and CSF collection is highly invasive and results take several weeks due to the lack of facilities that analyze CSF [17]. The downsides to these biomarker tests are that they are expensive, invasive, and can be painful, especially CSF tests [17,18]. 

Blood tests offer a more accessible and less invasive alternative to CSF biomarker testing. Current blood AD biomarkers include pathogenic AD proteins (Aß_40_, Aß_42_, T-Tau, and P-Tau), neurodegeneration markers (neurofilament light (NFL) and neurogranin), and inflammation markers (interleukin 1α (IL-1α), IL-1β, IL-6, IL-8, IL-33, intercellular adhesion molecule 1 (ICAM-1), progranulin, SDF-1, soluble interleukin 1 receptor-like (sST2), and vascular cell adhesion protein 1 (VCAM-1)) [17]. Proinflammatory biomarkers tend to be elevated in numerous diseases that are co-morbid with AD, such as depression, anxiety, heart disease, diabetes and inflammatory bowel disease (IBD) [1]. IL-1α, IL-1β, and IL-6, have been found to be altered in AD patients [17,20]. VCAM-1 and ICAM-1 plasma levels are also reported to be elevated in AD patients. VCAM-1 was similarly elevated in VD patients, and ICAM-1 was elevated only in AD compared to other non-inflammatory neurological diseases [21,22]. A relationship between IL-33 and sST2 serum levels has been suggested, as sST2 is elevated in AD patients, while IL-33 is downregulated [17,23].

A caveat to biomarker studies is the overlap in certain biomarkers between diseases and the high variability in blood expression levels within each disease. However, the combination of biomarkers that are differentially expressed and the specific blood levels for each biomarker appears to be different in AD compared to other co-morbid diseases (Table 1). For instance, studies of serum IL-6, ICAM-1, and VCAM-1 levels in AD patients and depression patients show some differences. Serum IL-6 levels were higher in AD patients than depression patients [24,25,26]. VCAM-1 serum levels also appeared to be greater in AD patients compared to depression patients [27,28]. There is controversial evidence on whether ICAM-1 serum levels differ between AD and depression [29,30,31]. Similarly, NFL may not be an AD specific biomarker as it was also altered in mild cognitive impairment (MCI) patients [17]. Studies have found that plasma NFL is elevated in AD patients and was able to differentiate *APOE ε4* carriers 16 years prior to expected symptom onset [17,32,33]. 

Biomarkers that are more specific to AD include proteins in the APP processing, tau and synapse formation pathways and are also differentially expressed in AD patients compared with healthy participants. While Aß_40_ levels in the blood do not detectably change, Aß_42_ levels decrease in single-molecular mass analysis (SIMOA) when CSF levels are pathological [17,34]. P-Tau levels are generally significantly lower in the blood than CSF, but have been found to be significantly elevated in AD patients [35]. Plasma neurogranin, a synaptic protein, is believed to be reduced in AD and FTD patients and may be detectable years before dementia onset [17,36]. 

**Table 1:** Blood levels of AD biomarkers in both AD and other co-morbid diseases. The blood levels of several biomarkers for AD and co-morbid diseases, including proinflammatory markers, neurodegenerative markers and markers from the pathways affected in AD, such as amyloid precursor protein (APP) processing, neurofibrillary tangle (NFT) formation and synapse formation, are described. Arrows denote blood levels relative to healthy control participants, and hyphens indicate unavailable data. Levels relative to controls are indicated by arrows, and absence of arrows indicates a lack of a change.

	Serum Levels (pg/mL)					
Biomarker	AD	Depression	Anxiety Disorders	Cardiovascular Disease	Diabetes	Inflammatory Bowel Disease (IBD)
TNFα	1.6 ± 1.4 [37] ↑	4.1 ± 0.5 [38] ↑	2.4 ± 0.9 [39] ↓	3.1 ± 3.4 [40] ↑	7.5 ± 2.5 [41] ↑	29.4 ± 0.2 [42]
IL-1α	89.2 ± 17.6 [43] ↑	3.3 ± 0.4 [38] ↑	70.3 ± 3.6 [44] ↑	-	0.9 ± 4.8 [45]	7.6 ± 61.5 [46] ↑
IL-1β	4.7 ± 2.1 [47] ↑	1.2 ± 0.2 [48] ↑	5.0 ± 2.3 [49]	1.7 ± 0.2 [50] ↑	3.0 ± 1.0 [45] ↑	3.8 ± 43.0 [46] ↑
IL-6	4.4 ± 5.1 [37] ↑	2.9 ± 0.1 [51] ↑	12.6 ± 2.4 [44] ↑	4.3 ± 3.5 [50] ↑	4.3 ± 2.6 [45] ↑	4.5 ± 4.1 [52] ↑
IL-8	35.0 ± 4.1 [53] ↓	8.9 ± 4.0 [54] ↓	44.6 ± 16.2 [44] ↑	47.8 ± 71.2 [50] ↑	6.5 ± 5.3 [45] ↓	5.0 ± 12.1 [55] ↓
sST2	27.5 ± 7.1 [23] ↑	9000± 3300 [56] ↑	-	420.0 ± 49.0 [57] ↑	160 ± 60 [58] ↑	50.3 ± 52.9 [59] ↑
IL-33	5.9 ± 5.5 [60] ↓	17.2 ± 5.6 [56]	635.8 ± 6.7 [61] ↑	103.3 ± 19.3 [62] ↓	40 ± 7 [63] ↓	40 ± 52.5 [64]
SDF-1	1949.6 ± 427.9 [65] ↓	4928.8 ± 589.5 [66] ↑	1352 ± 733 [67] ↑	1891.8 ± 1044.8 [68] ↑	204.2 ± 30.9 [69] ↑	-
progranulin	45.3 ± 11.8 [70,71] ↑	-	-	3.5 × 10^4^ ± 8.2 × 10^3^ [72]	47.2 ± 4.5 [73] ↑	-
VCAM-1	9.5 × 10^5^ ± 1.6 × 10^5^ [74] ↑	1.2 × 10^6^ ± 4.5 × 10^5^ [75] ↑	6.3 × 10^5^ ± 1.4 × 10^5^ [76] ↑	1.7 × 10^6^ ± 3.4 × 10^5^ [77] ↑	736.4 ± 267.0 [78] ↑	6.0 × 10^5^ ± 1.5 × 10^5^ [79] ↑
ICAM-1	3.4 × 10^5^ ± 3.2 × 10^5^ [80] ↑	2.7 × 10^5^ ± 8.7 × 10^4^ [75] ↑	2.4 × 10^5^ ± 4.3 × 10^4^ [76] ↑	1.6 × 10^6^ ± 3.6 × 10^5^ [77] ↑	245.4 ± 107.4 [81]	4.0 × 10^5^ ± 3.4 × 10^4^ [79] ↑
NFL	19 ± 12 [82] ↑	28.8 ± 22.5 [83] ↑	72.220 ± 22.8 [84] ↑	19.8 ± 12.2 [85] ↑	13 ± 4.5 [86]	-
neurogranin	429.2 ± 104.3 [87] ↓	100.3 ± 124.3[88] ↑	-	-	-	-
Aß42	44.2 ± 10.3 [37] ↑	11.4 ± 1.7 [89,90]	2.06 ± 0.2 [71]	-	-	-
Tau	351.9 ± 50.0 [91] ↑	4.3 ± 2.1 [90] ↑	0.27 ± 0.6 [71]	-	-	-

Blood tests of microRNA (miRNA) levels via reverse-transcription polymerase chain reaction (RT-qPCR) are another potential AD biomarker given their potential neurodegenerative and neuroprotective effects [17,92]. In the brain, studies have shown that about 50–70% of miRNAs are expressed and believed to play roles in normal brain physiology, including synaptic function and memory formation [17,93]. Table 2 lists miRNAs differentially expressed in AD patients. Given that many of the blood markers identified as potential AD biomarkers are not AD specific, it would be valuable to do combinational testing, potentially with MRI or another AD testing method, to rule out other neurodegenerative, neuropsychiatric and inflammatory conditions that can overlap with AD pathology and phenotypes. Some miRNAs regulate expression of genes in the Aβ pathway, including miR-22–3p and miR-340, both of which downregulate expression of BACE1, resulting in reduced Aβ formation [94,95,96]. In addition, miR-193a-3p and miR-148a-3p are protective against Aβ via regulation of JNK3 and ROCK1, respectively [97,98]. On the other hand, miRNAs, like miR-342–3p, increase expression of Aβ, exacerbating AD pathology [99].

Another AD diagnostic method gaining interest is using diagnostic imaging tests, such as optical coherence tomography (OCT), OCT-angiography (OCTA) and fundus photography. The retina is an extension of the brain that is easily accessible by non-invasive procedures and may demonstrate similar pathological changes to those observed in the brain during AD progression, such as the observed increases in Aß_42_ and Aß oligomers in the retinas of AD and mild cognitive impairment (MCI) patient post-mortem samples, with uneven distribution throughout the retinal layers [100]. Study of OCT in AD patients has also revealed reduced thickness in the peripapillary retinal nerve fiber layer (RNFL) and macula [17,101]. The downside of this test is differentiation from other conditions that also result in similar retinal pathologies, including multiple sclerosis (MS), glaucoma, and Parkinson disease (PD) [101]. OCTA is a technique that can visualize the retinal microvasculature at high resolution to detect changes in retinal vascular diseases. AD has been associated with vascular changes in the brain, which are believed to also appear in retinal capillaries. OCTA was used in advanced AD patients to demonstrate significantly reduced retinal capillary density, supporting this theory [102]. Fundus photography also revealed a more diminished vascular network, along with greater vein tortuosity [103]. A recent study examined the capillary pathology in asymptomatic carriers of the AD risk allele *APOE ε4* [104]. The researchers found that these asymptomatic carriers possessed reduced retinal capillary density compared to individuals that did not possess the risk allele, and this pathology corresponded with reduced cerebral blood flow in carriers assessed via MRI [104]. Pattern-electroretinogram (p-ERG) is another retinal procedure that has shown potential in early AD diagnosis. Reduced amplitudes were observed in mild cognitive impairment in AD patients and were distinguishable from VD patients [105,106]. The results of these retinal studies provide exciting insight into the potential of non-invasive retinal imaging as an early diagnostic tool for AD. However, the use of retinal imaging techniques for AD diagnostics is limited by overlapping phenotypes with ocular and other neurodegenerative diseases. While there are several approved testing options to potentially diagnose AD, the major difficulty for optimal treatment and intervention in disease progression is that these approved diagnostic tests are not performed until symptom onset, which can often not occur until the later stages of AD [107]. Better understanding of the changes that take place in the brain prior to and throughout AD progression is needed to improve the present diagnosis and treatment of AD.

## 2. Genetics of AD

**Figure 2:**
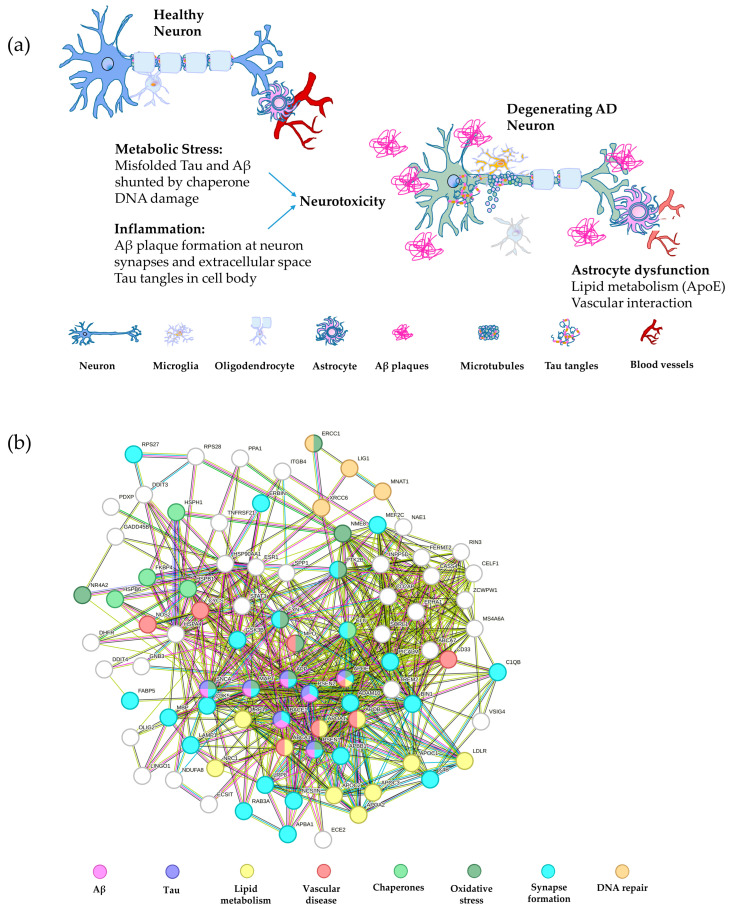
Schematic of major AD pathways and the associated gene network. (**a**) A schematic depiction of a healthy neuron compared with an AD neuron overlaid with the gene pathways leading to degeneration. The major pathways are inflammation, including Aβ and tau plaque formation, metabolic stress, such as misfolded tau and Aβ, in addition to DNA damage, and astrocyte dysfunction, involving abnormalities in lipid metabolism and vascular interaction with neurons [177,178,179,180]. (**b**) String analysis of the gene networks described in panel (**a**) color coded by pathway. The nodes represent proteins and the edges represent their functional associations (String v10) [115]. Interactions from curated databases are shown in blue and interactions mentioned in publications are given in green, while pink lines depict experimentally determined interactions and green lines represent proteins in close proximity.

AD is a complex disease with genetic and environmental influences. Presently, at least 34 genes with more than 100 variants have been identified as pathogenic or risk modifying in AD (Table 3, Table S1). Approximately 70% of AD cases are caused by mutations in *APP*, *PSEN1* or *PSEN2*, while the presence of the *APOE ε4* variant also confers risk [6,108,109,110]. AD is impacted by three major genetic pathways, which are amyloidogenesis, metabolism of lipids and tauopathy, while neurotransmitters and molecular chaperones also play a modifying role in AD pathogenesis (Figure 2, Figures S1–S8). Many of these gene pathways also play important roles in ocular diseases, such as age-related macular degeneration (AMD), diabetic retinopathy (DR) and glaucoma [111,112,113,114].

**Table 3:** Genes associated with AD. Associated genes include known AD genes and risk factor genes. Colored circles represent gene pathways associated with the gene using String Database [115]. Pink, Aβ pathway genes; blue, NFT pathway; yellow, lipid metabolism; red, vascular disease; light green, chaperones; dark green, oxidative stress; light blue, synapse formation; orange, DNA regulation and repair.

Gene and Pathway	Gene Name	Gene Function	AD Relevance from Mutations	AD Variants
*PSEN1 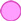 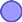 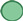 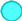 *	Presenilin 1	Encodes PS1 protein, a catalytic subunit of the γ-secretase enzyme that cleaves APP, resulting in Aß production [6,116]	Decreases Aß40 levels increasing Aβ42/Aβ40 ratio [6,109]	EOAD risk: 33 variants (Table S1)
*PSEN2 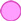 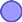 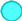 *	Presenilin 2	Encodes PS2 protein, a catalytic subunit of the γ-secretase enzyme that cleaves APP, resulting in Aß production [6,116]	Increases Aß42 levels increasing Aβ42/Aβ40 ratio [6,117]. Missense mutations rare cause of EOAD	EOAD risk: 6 variants (Table S1)
*APP 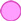 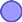 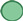 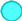 *	amyloid precursor protein	Encodes APP protein cleaved to release Aβ [6]	Promotes Aβ production/build-up and increases Aβ42/Aβ40 ratio [109]. Associated with familial EOAD	EOAD risk: 13 variants EOAD protective: 1 variant (Table S1)
*ECE2 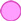 *	Endothelin-Converting Enzyme 2	Endothelin-converting enzyme/breaks down Aß	If ECE2 is not active, then it cannot breakdown Aß, leading to an excess in Aß	LOAD risk: c.556C>T c.2252T>C [118]
*GNB3 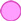 *	Guanine Nucleotide-binding protein, Beta-3	G protein β3 subunit/promotes adrenaline production	Different forms of the code coding for GNB3 can enhance APP expression	AD risk modifier: rs5443 [119]
*ADRB1 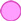 *	Beta-1-Adrenergic Receptor	β1-adrenergic receptor/promotes adrenaline production	Different forms of the code coding for ADRB1 can enhance APP expression	AD risk modifier: rs1801253 [119]
*CR1 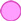 *	Complement component Receptor 1	Type-I transmembrane glycoprotein	Involved in eliminating Aβ and tauopathy [120]	AD risk: rs1408077 rs6701713 rs3818361 [121]
*SLC24A4/RIN3 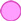 *	Solute Carrier family 24, member 4/Ras and Rab Interactor 3	Solute carrier	Increases endosomal dysfunction in APP/PSA1 mouse model [122]	Protective: rs10498633 rs12881735 [123,124,125]
*INPP5D 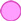 *	Inositol Polyphosphate-5-Phosphatase, 145-KD	Inositol polyphosphate-5-phosphatase family	Expression is elevated in microglia and associated with plaque in an AD mouse model [126]	Protective: rs61068452-G [127] LOAD Risk: rs1057258 rs35349669 [124,128,129,130]
*ECSIT 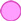 *	Evolutionarily Conserved Signaling Intermediate in Toll pathway	Encodes cytoplasmic/signaling adapting protein. Stabilizes mitochondrial respiratory complex [6]	Interacts with PSEN1, PSEN2 and APOE. Molecular link in AD inflammation, oxidative stress, and mitochondrial dysfunction [6,131]	
*CELF1 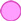 *	Cugbp- and Elav-Like Family, member 1	Alternate splicing of pre-mRNA	Affects expression of Aβ42	AD Risk: rs3740688 rs10838725 [132,133]
*FERMT2 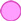 *	Ferm domain-containing kindlin 2	TGFβ1 receptor binding and actin binding	Involved in metabolism of APP [134]	Risk: rs7160582 rs7143400-T [128,135] Brain Amyloidosis: rs17125944 [136,137]
*CASS4 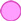 *	Cas Scaffold Protein Family, member 4	Tyrosine kinase binding	Possible role via regulation of CASS4 phosphorylation by α2β1 and αVβ1 integrins, which induces Aβ neurotoxicity [138]	Protective: rs7274581 rs6024870 rs6069736 [123] Pathogenic: rs16979934 [128]
*MAPT 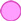 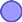 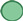 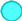 *	Microtubule-Associated Protein Tau	Encodes Tau protein/stabilizes microtubules	Tau tangles lead to destabilization of microtubules and death of neuron [139]	AD risk modifier: A152T [140]
*CD2AP 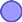 *	CD2-Associated Protein	Regulation of actin cytoskeleton	Loss causes neuronal toxicity resulting from tau [141]	LOAD risk: rs10948363 [142] rs9349407 [143]
*APOE 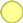 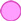 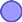 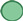 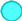 *	Apolipoprotein E	Metabolizes lipids and cholesterol [6]	*APOE ε4* allele increases AD risk while *APOE ε2* allele reduces risk. Role in formation of senile plaques from Aβ deposition. Associated with vascular damage and cerebral amyloid angiopathy [6,144,145]	EOAD and LOAD risk: c.127C>T [146]
*TREM2 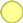 *	Triggering Receptor Expressed on Myeloid cells 2	Modifies microglia activity and survival	Increased expression in microglia cells surrounding amyloid	AD risk: rs75932628^T^ [147]
*ABCA1 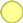 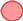 *	ATP-Binding Cassette, subfamily A, member 1	Regulates cholesterol transport from bloodstream into the brain. Stabilizes APOE lipidation and mediates HDL generation [6]	Increases Aß plaques and eliminates APOE lipidation. Decreases plasma HDL and ApoAI levels, cholesterol accumulation in tissues, and pathogenesis of AD [6]	AD possibly protective: P1059S V399A E1172D [148]
*ABCA7 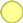 *	ATP-Binding Cassette, subfamily A, member 7	ATP-binding cassette transporter	Affects AD pathogenesis through regulation of lipid metabolism and clearing of amyloid [149]	Protective: rs3764650 rs72973581[149] LOAD Risk: rs4147914[128] AD Risk: rs3764650, rs4147929, rs3752246, rs115550680, rs78117248, rs142076058 [149]
*SORL1 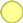 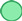 *	Sortilin-related Receptor	Participates in APP and Aß trafficking	Neurons without SORL1 show downregulation of APOE and CLU [150]	Protective: rs11218343 [123] LOAD Risk: rs2276412 [128]
*MPO 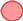 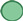 *	Myeloperoxidase	Inflammatory enzyme/catalyzes Cl and H2O2 to make HOCl, promotes production of reactive oxygen and nitrogen species	Over production of reactive oxygen species causes oxidative stress, which results in neuroinflammation [151]	EOAD risk: c.2031-2A>C c.1705C>T [146]
*CD33 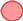 *	Sialic Acid Binding Ig-Like Lectin 3	Phosphatase and sialic acid binding activity	Short isoform leads to Aβ1–42 phagocytosis in microglial cells [152]	Risk: rs3865444-C rs12459419-C rs1803254 [128,153,154] LOAD protective: rs3865444-A rs12459419-T [153,155,156,157]
*CLU 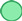 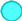 *	Clusterin	Lipid transport [6]	Promotes/Reduces Aß clearance [6]	LOAD Risk: rs1532278, rs9331947, rs11136000^C/T^, rs2279590, rs9331888, rs7012010, rs7982, and rs9331949 [128,136,143,158,159] Amyloid Deposition: rs3818361 [137]
*NME8 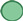 *	NME-NM23 family, member 8	Has a catalytically active N-terminal thioredoxin domain and implicated in ciliary function	Certain variants may play a role in reducing neurodegeneration [160]	LOAD Protective: rs2718058 [136,161]
*ESR 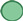 *	Estrogen Receptor	Binds estrogen	Implicated in neuroinflammation contributing to AD [37]	AD risk: rs6909023 rs2982684 [162]
*MS4A6A 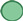 *	Membrane-Spanning 4-domains, subfamily A, member 6A	Membrane spanning protein	Over-expression increases neuroinflammation [163]	AD Protective: rs610932-A rs7232-T [164] LOAD Risk: rs12453 [128] Cortical/Hippocampal Atrophy: rs610932 [137]
*BIN1 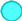 *	Bridging Integrator 1	Membrane curvature and endocytosis functions [6]	Participates in Aβ production and modulator of tau and NFT pathology [6,165]	LOAD risk: rs754834233 rs138047593 [166]
*ADAM10 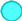 *	A Disintegrin and Metalloproteinase Domain 10	α-secretase/involved in cutting of APP ectodomain	Certain variants increase Aß levels *in vitro* and makes APP produce Aß in Tg2576 mice	LOAD risk: Q170H R181G [167]
*PTK2B 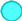 *	Protein-Tyrosine Kinase 2, Beta	Tyrosine kinase	Plays a role in Aβ-mediated synaptic defects [168]	LOAD Risk: rs4732720 rs28834970 [128,169]
*MEF2C 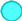 *	Myocyte Enhancer Factor 2C	Member of the MADS box transcription enhancer factor 2 family, plays a role in myogenesis	Knockdown in AD mouse model leads to elevated Aβ levels, downregulation of synaptic proteins and oxidative stress [84]	Protective: rs190982 [170] LOAD Risk: rs9293505 [128]
*PICALM 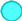 *	Phosphatidylinositol-binding Clathrin Assembly protein	Clathrin assembly	Down-regulated in AD brain correlating with autophagy defect [171]	LOAD Risk: rs7480193 rs510566 rs1237999 rs561655 rs17148741 rs3851179 [128,137,143,172]
*EPHA1 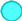 *	Ephrin receptor A1	Protein tyrosine kinase	Affects neuroinflammation [173]	AD protective: rs11762262 rs11771145 [123,136,174] LOAD Risk: rs11767557 rs11768549 [128,143]
*ZCWPW1 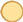 *	Zinc finger CW-type domain and PWWP domain-containing protein 1	Involved in the histone methylation process; possible role in meiosis I	Proposed to play a role via regulation of DNA and via reduction of insulin resistance	LOAD Protective: rs1476679 [132,175]
*HLA-DRB1/DRB5*	Major histocompatibility complex, class II, DR Beta-1/Beta-5	Human leukocyte antigen complex proteins	May be involved in AD pathogenesis through its role in the immune system	LOAD Risk: rs6597017 rs9271192 [128,176]

### 2.1. Amyloid-β Aggregation

**Figure 3:**
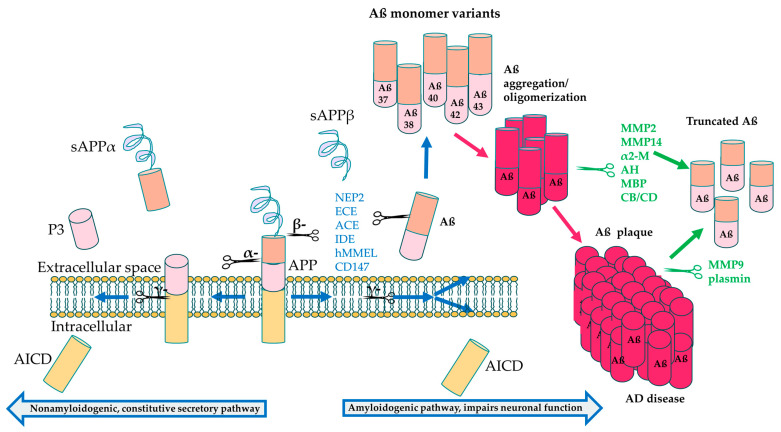
APP processing and cleavage leading to amyloid-β production. Proteolytic cleavage by α-secretase in the intracellular domain leads to formation of soluble APP α (sAPPα), peptide 3 (P3) and the APP intracellular domain (AICD) components, preventing Aβ aggregation. On the other hand, cleavage by β-secretase followed by cleavage by γ-secretase leads to formation of the neurotoxic Aβ42 monomers that aggregate to form amyloid plaque [182]. Aβ degrading enzymes interfere both in the healthy physiological state before Aβ oligomerization (blue text) and after aggregation (green text) to break down Aβ, resulting in less toxic, truncated variants, including Aβ20 and Aβ30 [185].

Aβ accumulation is often considered the most significant contributor to AD pathogenesis [6]. APP is a transmembrane protein that normally undergoes proteolytic cleavage by α-secretase that results in soluble APPα formation, which has numerous physiological roles, including neurogenesis, synapse formation and sequestering of metal ions [181]. However, in the pathological state, β-secretase cleaves APP, along with subsequent cleavage by γ-secretase, resulting in formation of insoluble Aβ monomers that oligomerize and eventually aggregate into plaques [182]. The amyloid accumulation pathway includes *PSEN1*, encoding a subunit of γ-secretase, which is the most common gene associated with EOAD and has the greatest number of pathogenic mutations, followed by amyloid precursor protein (*APP*) and *PSEN2;* mutations in these AD genes are associated with the build-up of Aß peptides in the brain. *PSEN1* and *PSEN2* are involved in the γ-secretase complex responsible for cleavage of the APP protein to make Aß peptides, as shown in Figure 3 [183]. Mutations in *PSEN1/2* lead to increased cleavage of APP into the Aß_40_ and Aß_42_ subunits, which induces the formation of insoluble amyloid fibrils [17]. 

Several protein classes are known to degrade Aß monomers and plaque in the normal brain, and these include the metalloproteinases and the aminopeptidases, such as serine proteases, aspartate proteases, as well as cysteine and threonine proteases [184,185]. These can be further subdivided into endogenous and pathological proteases, with endogenous proteases expressed under homeostatic conditions, and these include insulin-degrading enzyme (IDE), neprilysin (NEP) and endothelin converting enzymes (ECE). On the other hand, plasminogen is converted to plasmin in the presence of Aß plaque build-up and is active under pathological conditions. Some of these proteins, including NEP, IDE and ECE, break down Aß monomers in the healthy physiological state, while some degrade Aß oligomers and fibrils, such as MMP2, MMP14, acylpeptide hydrolase (AH), as well as cathepsin B (CB) and cathepsin D (CD) (Table 4). MMP9 and plasmin can also degrade compact plaque in addition to fibrils and monomers [185,186], making these prime candidates as therapeutic targets for AD. Proteins that degrade Aß oligomers could also potentially be developed into therapies, since a recent study suggested that soluble Aß oligomers may be more toxic than plaque formation [187].

Build-up of amyloid fibrils accumulating into Aß plaques that is not broken down by Aß degrading proteins leads to neurotoxicity and blocked neuronal communication in AD [6,188]. Recent evidence suggests that Aβ aggregation can increase DNA double stranded breaks (DSBs) in neurons [178,180,189], which could be a potential mechanism of neurotoxicity. The accumulation of Aß is also suggested to induce the other pathologies seen in AD, including tau pathology [17]. Interestingly, approximately 14% of patients with mild to moderate AD do not have any or very sparse Aβ plaque [190], suggesting that other gene pathways also contribute to AD pathology.

**Table 4:** Aβ degrading enzymes. Aβ degrading enzymes fall under the metalloproteinases and aminopeptidases that either break down Aβ monomers, fibrils or compact plaques [184,191,192]. MMP, metalloproteinase; NEP, neprilysin; ECE, endothelin converting enzyme; ACE, angiotensin converting enzyme; IDE, insulin-degrading enzyme; α2-M, α-2 macroglobulin; AH, Acylpeptide hydrolase; MBP, Myelin basic protein; ACT, α1-antichymotrypsin; CB, Cathepsin B; CD, Cathepsin D; BACE, β-secretase.

Enzyme Type	Enzyme	Substrate	Significance in AD
Metalloproteinase	MMP2	Aβ fibrils	Expressed in the healthy state
	MMP9	Aβ fibrils and Compact plaques	Expressed in the healthy and pathological states
	MMP14	Aβ fibrils	Expressed in the healthy and pathological states
	NEP	Synthetic Aβ oligomers	Expressed in healthy and pathological states
	NEP2	Aβ monomers [188]	Expressed in healthy physiological state
	ECE1	Aβ monomers	Expressed in healthy physiological state
	ECE2	Aβ monomers	Expressed in healthy physiological state
	ACE	Aβ monomers	Expressed in healthy physiological state
	IDE	Aβ monomers	Expressed in healthy physiological state
	hMMEL	Aβ monomers	Expressed in healthy physiological state
	CD147 [193]	Aβ monomers [194]	Expressed in healthy physiological state
	α2-M [195]	Aβ fibrils	Overexpressed in pathological state
Aminopeptidases	plasmin	Aβ monomers, fibrils and compact aggregates	Expressed in the pathological state
	AH	Aβ oligomers	Expressed in the pathological state
	MBP	Aβ fibrils	Expressed in healthy physiological state
	ACT	Aβ monomers	Increases Aβ polymerization
	CB	Aβ fibrils	Expressed in healthy physiological state
	CD	Aβ fibrils	Expressed in healthy physiological state
	BACE1	APP	Expressed in healthy and pathological physiological states
	BACE2	APP	Expressed in healthy and pathological physiological states
	Proteasome	Aβ monomers	Expressed in healthy physiological state

### 2.2. Tau Accumulation

The second major pathway affected in AD is the tau pathway. Tau protein, encoded by the microtubule associated protein tau (*MAPT*) gene, is a neuronal protein normally found in axons and is important for the stabilization of microtubules. Pathogenic variation leads to hyperphosphorylation of tau [196], rendering the protein unable to bind to the microtubules. This leads to disassembly of microtubules, which makes the cell lose its organization [17,197]. Hyperphosphorylated tau then accumulates in neurons and leads to NFT formation, characteristic in AD pathology, which can result in neuronal death and reduced movement of APP in the axon (Figure 1e) [198]. Tau is also proposed to be in a feedback loop with Aß in AD, with initial Aß accumulation suggested to increase formation of NFTs that further enhances Aß aggregation [196,198]. Both Aβ and tau plaque formation are linked to AD pathology; however, more recent studies demonstrate the involvement of other pathways that also overlap with ocular and neurodegenerative diseases.

### 2.3. Lipid Metabolism

The third main pathway affected in AD is lipid metabolism, and this pathway includes the *APOE* gene, which possesses three isoforms (ApoE2, ApoE3, and ApoE4) [6]. In the brain, *APOE* is expressed mainly in astrocytes and activated microglia, and plays a role in lipid and cholesterol transport to neurons, and in the formation of synapses, neurite outgrowth and tissue repair [199]. *APOE* is frequently associated with sporadic LOAD risk [17,200,201]. The *APOE ε4* allele increases the risk of AD through Aß build-up, whereas *APOE ε2* reduces risk [202], while the *ε3* variant has an intermediate phenotype [6]. The *APOE ε4* isoform is associated with reduced efflux of cholesterol, potentially increasing lipid accumulation in astrocytes and microglia [203,204,205] that could be contributing to AD pathology. The *APOE ε4* isoform has been shown to stabilize Aβ oligomers [206,207], which could contribute to elevated AD risk. Excess Aß leading to brain effusion or hemorrhage is also more common in those who have a variant in the *APOE* gene that confers AD risk [208]. Moreover, weaker binding affinity of the APOE *ε4* variant for phosphorylation sites on the tau protein [209,210] may potentially explain the increased deposition of tau in AD patients [211]. *APOE* and lipid metabolism pathways overlap with retinal diseases, including maculopathies, such as AMD. Incidentally, the *APOE ε2* allele that is protective in AD confers risk in AMD, whereas *APOE ε4* is protective in AMD [111], highlighting the complex roles of these gene pathways in different neurodegenerative diseases.

### 2.4. Acetylcholine

In addition to Aß and tau, an important molecule considered crucial in cognitive function is the neurotransmitter, acetylcholine (ACh) [6]. It is produced in the cytoplasm of cholinergic neurons by the enzyme choline acetyltransferase (ChAT) and is transported by vesicular acetylcholine transporter to the synaptic vesicles [6]. ACh is used in many processes in the brain, including attention, learning, memory, and sensory information [6]. Aß neurotoxicity and interactions between Acetylcholinesterase (AChE) and Aß lead to progression of neurodegeneration through cholinergic synaptic loss, and amyloid fibril formation [6,212,213], which negatively affects cognitive performance and memory in AD.

### 2.5. Chaperones

Chaperones are garnering interest among the AD research community as potential targets for therapies, since some chaperones have been shown to either increase protein aggregation or reduce it, particularly in the amyloid aggregation and NFT formation pathways. Molecular chaperones are proteins that assist with correct protein folding or target misfolded proteins for degradation, and are expressed in response to cell stress, such as extreme heat, ischemia, oxidative stress, heavy metal stress and alcohol [214]. Chaperones play a role in protein homeostasis by assisting with de novo protein folding and refolding of misfolded proteins and preventing abnormal interactions between intermediately folded proteins. Protein aggregate diseases result when these homeostatic processes are disrupted [215]. The chaperones, Hsp20, Hsp27 and αB-crystallin, can prevent formation of Aβ aggregates in a cell culture model of cerebrovascular neurotoxicity, which is observed in VD [216,217]. Another chaperone protein, clusterin, shows mixed results, with some studies showing that clusterin can bind oligomeric Aβ species and stabilize them, reducing uptake of Aβ by neurons [218], while others show that clusterin increases uptake [219,220,221]. Chaperones can regulate tau aggregation both in vitro and in vivo. 

Chaperones also affect the tau pathway, with HSP70 previously shown to reduce aggregation of wild-type tau protein in vitro. These findings extended to in vivo animal studies showing that HSP70 and its co-chaperone, HSP110, were required for preventing hyperphosphorylation of tau [222,223]. On the other hand, HSP90, when co-expressed with FKBP prolyl isomerase 5 (FKBP51), leads to reduced clearance of tau monomers and formation of tau oligomers, causing neurotoxicity, in a mouse model [224]. Recent studies demonstrate that chaperones could provide a neuroprotective role in response to oxidative stress resulting from mitochondrial damage occurring in AD neurons [178,225,226,227], and this protective role can be harnessed for developing therapies.

## 3. Environmental Risk Factors

There are many different environmental factors that put many people at risk of developing AD, including older age, pollutants, metals, neurotoxins and brain injury, while some factors may have a protective effect, including a healthy diet and some gut microbe species. The most prevalent and well-known cause of AD is aging. As normal aging progresses, the chance of developing AD increases with the natural degeneration of the body, including cognitive decline, reduced brain volume, and synaptic loss. AD is an accelerated version of the natural aging process [212,213]. 

### 3.1. Air Pollution and Heavy Metals

Some AD risk factors are less prevalent, such as air pollution. It was found that people who are exposed to polluted air can develop neurodegeneration, neuroinflammation, and oxidative stress [228]. Metals can also be a factor, as aluminum has been found to accumulate in the brain, specifically the hippocampus, the cortex, and the cerebellum [229]. It interacts with proteins such as APP and APOE, causing them to unfold. Lead can compete with calcium in binding sites and cross the blood–brain barrier (BBB) where it can affect the synthesis of neurons and synapses [230]. Lead is also associated with a build-up of Aß [231]. Cadmium is another metal that can cross the BBB and cause the build-up of Aß plaque and tau, which can lead to AD [232]. These studies suggest that reducing exposure to high levels of air pollution and heavy metals can potentially lower the risk of developing AD.

### 3.2. Diet and the Gut Microbiome

Emerging research shows the important role of the gut microbiome in AD. In the stomach and throughout the body, there are thousands of microorganisms that affect our development and physiology. The microorganisms that make up the gut microbiome can be bacteria, fungi, and viruses. Probiotics are the microbes that positively impact our body by defending against diseases and reduce inflammation [233]. The connection between the gut microbiome and AD lies in the connection between the gut microbiome and the brain. The GI tract contains the enteric nervous system (ENS) that forms the gut–brain axis via the central nervous system (CNS) and blood vessels.

Microbes can produce substances, such as neurotransmitters and neuromodulators that can interact with neurons in the CNS, which can have various effects on a person’s behavior and cognition. The *Lactobacillus* and *Bifidobacterium* species commonly found in the human gut can convert glutamate to Gamma-amino butyric acid (GABA), an inhibitory neurotransmitter that plays a role in cognition, with dysregulation associated with defective synapse formation and cognitive impairment in AD [234,235]. Interestingly, *Bifidobacterium* levels were reduced in AD patients compared with control participants [236,237]. In addition, some *Bacillus* species can produce acetylcholine, another neurotransmitter perturbed in AD [238], while *Lactobacillus*, *Bifidobacterium* and *Clostridium* species can produce short chain fatty acids (SCFAs) [239] that are capable of ameliorating Aβ plaque formation [240]. The *Bacteriodes*, *Alistipes*, *Barnesiella*, and *Odoribacter* species were found to be associated with preclinical AD, while *Methanobrevibacter smithii* had a negative correlation with levels of butyrate, an SFCA [236,237,240].

Microbes can also produce neurotoxins that can increase AD risk. Cyanobacteria in the gut has been proposed to generate β-N-Methylamino-L-Alanine (BMAA), a neurotoxin elevated in AD [241]. Certain metabolic byproducts, such as ammonia and d-lactic acid, can also exert neurotoxic effects due to increased permeability of the gut and the blood–brain barrier resulting from inflammation in aging [242]. In particular, ammonium can lead to formation of Aβ_42_ plaques in the astrocytes in a murine primary cell culture model [243]. Inflammation can also cause microbes, like *Escherichia* and *Shigella* species, to produce proinflammatory cytokines elevated in AD, such as IL-1, IL-6, tumor necrosis factor-alpha (TNF-α) and transforming growth factor-beta (TGF-β), which can change behavior and induce anxiety, depression and memory loss [244]. On the other hand, the presence of *Eubacterium rectale* had an anti-inflammatory effect [244,245]. These studies show that an individual’s specific microbiome profile plays a complex role in AD pathophysiology and is modifiable through lifestyle choices, such as diet.

### 3.3. Pre-Existing Conditions

Pre-existing brain defects can affect a person’s likelihood of developing AD. Brain trauma resulting from traumatic brain injury (TBI) can cause some neurons in the medial temporal lobe to secrete large amounts of APP. The large amount of APP then causes a build-up of Aß. It has also been discovered that chronic traumatic encephalopathy (CTE) is directly related to brain injury. CTE is a very similar disease to AD in that they are both neurodegenerative diseases that result in impaired memory, loss of motor function, and behavioral changes/changes in mood. The tau phosphorylation in CTE is also similar to AD [246], suggesting that CTE may increase risk for AD.

It has also been shown that people afflicted with Down Syndrome have a 90% risk of developing dementia, with Aß plaques and NFTs forming by 40 years old [247], and they have an 88–100% chance of developing AD after the age of 65. In addition, 95% of people with Down Syndrome have a third copy of chromosome 21, and this third copy causes the *APP* gene to triple, which causes an excess in Aß, leading to early-onset dementia [248]. Studying how pre-existing conditions, like CTE and Down Syndrome, contribute to AD risk can lead to greater understanding of the molecular mechanisms underlying AD.

## 4. Models of AD

### 4.1. AD Modeling in Humans Using Single-Cell Genomics

Single-cell genomics have contributed to our understanding of AD by giving a finer resolution of the transcriptional and epigenetic states of each part of the brain and how it changes over the course of AD. Briefly, single-cell (or nucleus) RNA-seq labels the RNA that belongs to each cell with a barcode, which can be deconvoluted and clustered to identify which cells (or nuclei) belong to which cell type in the brain. To date, over 3300 cell subclusters in the human brain [249] and 1000 cell supertypes in the mouse brain have been identified [250].

Not only RNA and transcription can be assessed at the single-cell level. Complementary to RNA, single-cell ATAC-seq (Assay for Transposable-Accessible Chromatin) [251] provides a readout of what DNA is not bound up in chromatin and is therefore available to be actively transcribed into RNA. ATAC-seq first cuts accessible DNA and barcodes it, then the open DNA chromosomal locations are clustered and assigned a cellular identity. The two paradigms of single-cell RNA-seq and single-cell ATAC-seq can be paired to provide a more accurate view of the open chromatin states preceding transcription, and the deciphering of gene regulatory networks focused on transcription factors and their downstream targets [252]. Coupling RNA with spatial information, that is, using a variety of techniques to identify mRNA expression at different positions of a tissue slice, offers even greater opportunities to resolve where cell types come from [253].

Several seminal studies have elucidated the underpinnings of AD in the brain at single-cell/nuclei resolution. By profiling the prefrontal cortical area of human AD and undiagnosed healthy control samples at a single nuclei level, Lau et al. (2023) found the processes of angiogenesis, immune activation, synaptic signaling, and myelination to be disrupted in the AD brain. At this granularity, endothelial cells, astrocytes, and oligodendrocytes were shown to be the cell types most affected in these processes [254]. 

In AD and other neurodegenerative diseases, breakdown of the BBB, which protects the neurons from circulating factors and pathogens, is implicated. As such, neurotoxic infiltrants are able to enter, and there is an inflammatory and immune response [255]. Focusing specifically on the vasculature, Yang et al. (2022) developed a special protocol to extract single nuclei for RNA-seq from vessels, supporting astrocytes, and immune cells from healthy undiagnosed control subjects and AD patients. At this resolution, specific subpopulations of the vasculature, particularly from endothelial cells and pericytes, which are the major components of the BBB, were down-regulated in AD [256].

Following up on these works, Mathys et al. (2023) created an AD specific single-cell atlas of subjects with varying degrees of AD pathology and cognitive decline. From this work, they revealed the types of inhibitory neurons most depleted in AD, as well as those inhibitory neurons that, when abundant, result in higher cognitive function [177]. Studies of brain samples from living patients with AD also revealed signatures specific to early stage-disease, including an increased ratio of excitatory to inhibitory activity in the parietal cortex and temporal cortex, increased expression of TGF-β pathway components important for Aβ clearance in microglia, and upregulation of amyloidogenic genes in oligodendrocytes and excitatory neurons [257].

Morabito et al. (2021) conducted a study combining single-cell resolution RNA and ATAC sequence to evaluate late-stage AD patients and healthy age-matched controls. Their multi-omic integrative analysis revealed a number of cell-type specific, cis-acting gene regulation changes in astrocytes, inhibitory neurons, excitatory neurons, microglia, oligodendrocytes, and oligodendrocyte precursor cells [258]. Gamache et al. (2023) also paired late-onset AD and matched healthy controls with multi-omics approaches. Similarly, they found cell-type specific cis-acting gene accessibility networks [259]. As more single-cell studies emerge, we will learn more about AD disease pathology at the level of detail of the cell type, and how each is affected by AD. Leveraging RNA, DNA, and spatial information at a single-cell level will create a fuller picture as to how the brain changes in AD pathology. Which cell types interface with the amyloid-β plaques, and indeed if there are any compensatory mechanisms to try to overcome the disease burden, will become clearer.

### 4.2. Animal Models of AD

Several animal models of AD have been developed, including both vertebrate models, such as non-human primates (NHP), canine models, murine models and the zebrafish, as well as invertebrate models, such as *Drosophila* models (fruit flies) and the *C. elegans* models (Table 5). Each type of model offers distinct advantages, with some, like NHPs, representing human AD pathophysiology more faithfully through spontaneous development of the disease, while others, like murine, zebrafish and invertebrate models, involve ease of induction or genetic manipulation.

#### 4.2.1. Non-Human Primate Models

Although NHP and canines can develop AD, it is not naturally occurring in most other animals. AD can take years to decades to develop naturally in NHP models, such as apes, rhesus macaques, and baboons. In NHPs, there is usually Aß plaque build-up, with tauopathy being rare, while humans can develop both [260]. Very few studies have been carried out on NHP models with naturally occurring AD, since senile plaque formation occurs in aging primates usually past the age of 30. A study using senile rhesus macaques has shown a correlation between cognitive decline and Aß build-up similar to AD in humans [261,262]. Another study using stump-tailed macaques yields similar results, although at an older age [263]. Formaldehyde also accumulates in excess in aging rhesus macaque models, as seen in human AD patients [264]. Aß plaques in NHP models are seen to accumulate in different places than in humans. In humans, Aß plaques are found in the hippocampus, amygdala, olfactory cortex, frontal cortex, temporal lobe, and parietal lobe. In rhesus macaques the plaques develop in the marginal cortex and prefrontal lobe [265]. Since NHPs do not exactly recapitulate the human phenotype, this results in the need to artificially induce AD in NHP models. One induced model was created by injecting Aß oligomers into the NHP model to cause AD-like symptoms [266]. Macaque monkeys that were injected exhibited Aß accumulation, tau hyperphosphorylation and glial activation. In another study using rhesus monkeys, AD pathologies, like Aß build-up, cholinergic neuronal atrophy and glial activation, occurred 7 weeks after injections of Aβ and thiorphan (an inhibitor of a protein that disposes of Aß) [263,267]. While the use of NHP models is still valuable, the costs of using models with naturally occurring AD outweighs the benefits.

#### 4.2.2. Mouse/Rat Models

Most of the AD animal models studied are transgenic mouse models. Currently, there are several rodent models of AD, including more than 11 mouse models and 4 rat models [263]. Murine models mainly have mutations in the *APP*, presenilin and *MAPT* genes, with various combinations of mutations in the different models. Mouse *APP* is 97% similar in structure to human *APP* and, among the *APP* isoforms that are different between the species, 3 (R5G, Y10F and H13R) hinder the accumulation of Aß [268]. One potential AD mouse model has the Indiana mutation driven by PDGF-ß [269] and expresses the human wild-type APP, which increases the production of Aß; however, it does not show any association with AD. This model also has an over-expression of APP, leading to Aß plaque build-up in the cortex and hippocampus, synaptic impairment, and loss in cognitive function [270,271]. Another AD model is a mouse model with a FAD (familial AD) mutation, the Tg2576 mouse, which expresses *APP* with the double Swedish mutation driven by the PrP promoter. This mutation also causes the over-expression of *APP*, and these mice develop Aß plaques in frontal, temporal and entorhinal cortices, the hippocampus and cerebellum, along with a similar phenotype to the PDAPP mouse [272]. The APP23 mouse also expresses the double Swedish mutation but driven by the Thy1 promoter. This model has immediate plaque development and more targeted neurodegeneration [273], whereas Tg2576 mice have more spread-out plaques. The J20 mouse has both the Indiana and Swedish mutations and shows more severe results, indicating that adding more mutations to *APP* adds more severe effects [274]. The 5xFAD mouse with five mutations in the *PSEN1* and *APP* genes is also widely used as a model for AD [275,276].

Wild-type mice do not produce tau tangles due to differences in the sequences of mouse and human tau proteins. Mice only express 4R isoforms, whereas humans express a mix of 3R and 4R [277]. Expression of human tau in mice only causes tangles when the mouse has no intrinsic tau, and NFTs only form in mice when human tau with a mutation associated with frontal and temporal lobe degeneration is expressed [278]. The JNPL3 mouse with human 0N4R tau expressing the P30L1 mutation in the *MAPT* gene shows motor deficits accompanied by tangles in the diencephalon and spinal cord [279,280]. Similar to this mouse, the rTg4510 mouse expresses the P30L1 mutation but under control of a tetracycline responsive element, and displays inclusion bodies in the cortex and hippocampus, along with neuronal loss and cognitive decline [281]. The PS19 mouse is another model for tauopathy expressing the human 1N4R tau isoform with the P301S mutation and exhibiting NFT formation in the cortex and hippocampus, in addition to neurodegeneration and activation of microglia [282]. Finally, there are the models which produce both tau tangles and Aß plaques. These models have mutations in the *APP* and *MAPT*, sometimes *PSEN1* or *2*, such as the 3xTg, which develops Aß plaques first at 6 months and then tau tangles after 12 months. These mice experience minor neurodegeneration with limited production of Aß and tau that does not appear as in sAD (sporadic AD). Widespread tangles and plaques are not apparent until later in life [283]. Similar to the 3xTg model, the pR5-183 mouse model also has mutations in both tau and Aβ pathway genes, including a P301L tau mutation, as well as the PS2N141I and APP_swe_ mutations [284]. This model was studied at the single-cell level, revealing much about the transcriptional stage of the disease. Zeng et al. (2023) joined single-cell transcriptomics with spatial information in a protocol they developed and found that disease-associated microglia are in close contact with the amyloid-β plaques [284]. However, disease-associated astrocytes and oligodendrocyte precursor cells are along the outer shell, providing key spatial information that corresponds to the transcriptional changes [284]. There are also lesser used models, like octodon degu, which develop Aß plaques and tau build-up, leading to AD like symptoms [285].

Rats have been used to model AD, although to a lesser extent, due to limitations in availability of genetic manipulation techniques. These models include Tg478/Tg1116, with the Swedish/London mutations and the hAPP695 mutation showing amyloid plaques by 17 months, as well as the PSAPP model, with Tg478/Tg1116 and presenilin mutations showing amyloid plaque by 9 months [286,287]. The McGill-R-Thy1-APP rat with the Swedish, Indiana and hAPP751 mutations and the TgF344 model having the hAPP695 mutation are both models that show amyloid plaque formation during aging, with the latter model also displaying Gallyas-positive structures resembling NFTs [288,289]. The ease of genetic manipulation in rodents is offset by lower translatability compared to NHPs; however, they contribute a great deal to the understanding of AD pathophysiology.

#### 4.2.3. Canines

Canines are another model used to study AD. Dogs can develop a disease called canine cognitive dysfunction (CCD) or canine dementia that is very similar to human AD, which is one of the advantages of using a canine model, since other species do not recapitulate the cognitive decline component of human AD [290]. Aß in dogs also has the same peptide sequence and it accumulates in a very similar fashion. Tau tangles, however, do not accumulate in canines [263]. Dogs with CCD also show atrophy in similar brain regions as humans, like the cortex and hippocampus [291]. In addition to brain atrophy, dogs with CCD also exhibit similar cognitive symptoms as AD patients, such as confusion, anxiety, timidity and poor recognition of owners [292]. Despite the similar cognitive phenotype as human AD, the low availability of canine models and the associated costs limit their use.

#### 4.2.4. Zebrafish Models

Zebrafish models of AD have many genes orthologous to the human counterparts implicated in EOAD, including *PSEN1*, *PSEN2* and *APP*, in addition to other genes involved in AD generally, such as *APOE* and *MAPT,* which makes zebrafish good candidates for performing gene mutation studies. Zebrafish models with *psen1* or *psen2* mutations resulting from morpholino injections showed similar defects to *Psen1*^−/−^ and *Psen2*^−/−^ mice [293,294,295], including Notch pathway signaling deficits in both zebrafish mutants and aberrant development of somites in *psen1* mutants [296,297]. Mutations in the zebrafish *app-b* gene resulted in abnormalities in synapse formation that could be rescued with delivery of human full-length APP protein [298]. There also exist orthologs for genes involved in the γ-secretase pathway, allowing for modeling of AD pathological pathways [299], including hypoxia, a condition that is easier to control in aquatic species. Moreover, zebrafish models are often used for drug screening, since compounds can easily be mixed into the water [300]. Zebrafish also show behaviors that can be used as quantifiable measures of cognitive function relevant to AD symptoms, such as avoidance and lack of startle response habituation [301]. The low costs compared with other animal models and ease of breeding make zebrafish a good animal model for studying the effect of specific gene mutations.

#### 4.2.5. Invertebrate Models

Similar to zebrafish, there exist orthologs for AD genes in invertebrate species, such as *C. elegans* worms and fruit flies [302]. The *C. elegans* contains genes that have human homologs, including AD genes. These worms also show conservation of synaptic mechanisms and genetic pathways with humans. Transgenic production of Aβ_42_ in the *C. elegans* muscle led to Aβ accumulation and paralysis, which was then used to model oxidative stress in AD [302]. In multiple *C. elegans* models, each expressed Aβ along with different human *APOE* variants, showing that *APOE ε2*, a protective allele against human AD, was associated with reduced degeneration of glutamatergic neurons. On the other hand, worms expressing the human *APOE ε4* variant, which increases AD risk, showed absence of protection against neurodegeneration caused by Aβ [303].

Fruit flies also have genes orthologous to human AD genes, such as presenilin, *APP*, and tau, and exhibit certain behaviors similar to mammals, including attention, learning and memory, and aggression, which can be used to measure the effect of genetic manipulation [263]. A fruit fly model with *psen* mutations affecting the γ-secretase pathway showed abnormal synapse formation and impaired learning [304], while a model with a tau mutation showed neurofibrillary pathology [305], and another model with an Aβ mutation showed Aβ plaque formation and neuronal cell death [306]. Transgenic expression led to ocular defects in another fruit fly model of AD, while also leading to reduced lifespan [302]. These models highlight the ease of genetic manipulation and of measuring simple behaviors that reflect these genetic changes in these invertebrate models.

## 5. Current Treatments

### 5.1. Donepezil

Acetylcholine is implicated in cognition, with deficits in cholinergic pathways observed in AD [307]. Several inhibitors of cholinesterase, which metabolizes acetylcholine, have been developed that, although do not reverse AD, treat symptoms associated with AD. Donepezil is a cholinesterase inhibitor that is FDA approved for treating cognitive decline in mild to moderate AD, and has been shown in several clinical trials to improve signs of dementia in AD patients [307]. In particular, two studies showed improvements in the CDR-SB test and the MMSE test outcomes [308], as well as in facial and name recognition [309] in AD patients when compared to the placebo group. AD patients also showed a smaller reduction in volumes of the entire hippocampus and the right hippocampus compared with the placebo group in another study [310].

### 5.2. Rivastigmine

Rivastigmine is another cholinesterase inhibitor drug, FDA approved to treat dementia associated with mild to moderate AD. One particular study showed that AD patients treated with the high dose showed 24% improvement in the cognition subscale of the AD assessment scale by at least 4 points, compared with 16% in the placebo group. Global function was also found to improve in the high dose group compared with the placebo group, and importantly, scores on the progressive deterioration scale improved when compared with baseline in the high dose AD patient group, whereas this score decreased in the placebo group [311], while another study replicated similar findings [312].

### 5.3. Galantamine

Galantamine is another FDA approved cholinesterase inhibitor for treatment of dementia. Clinical trials have shown the efficacy of galantamine in improving scores on the cognitive subscale of the AD assessment scale, as well as on the progressive deterioration outcome scale (PDS) [313]. Another study also showed improvements in global outcomes, as well as activities of daily living (ADL), in AD patients when compared with the placebo group, but there was no change in behavioral outcomes as assessed using the Neuropsychiatric Inventory (NPI) [314], with other studies replicating these findings. Interestingly, data shows an absence of effect of the *APOE* risk variant on cognitive outcomes in patients receiving galantamine [315].

### 5.4. Memantine

*N*-methyl d-aspartate (NMDA) antagonists are used for the symptomatic treatment of AD. NMDA receptor is proposed to have a role in AD pathology by triggering an influx of Ca^2+^ leading to synaptic dysfunction and cell death [17,316]. Memantine is the only FDA approved NMDA antagonist and is recommended for moderate to severe AD. It is a type of glutamate receptor and a low-affinity uncompetitive antagonist that functions by preventing over-activation of glutamate receptors [17,316]. Previous studies conducted to evaluate the efficacy of memantine demonstrated improved cognitive and behavioral outcomes, reduced patient stress and reduced caregiver burden for treated patients compared to placebos [317,318]. Other NMDA antagonists tend to increase the risk for developing schizophrenia-like symptoms; however, this was not the case with memantine [319], which may even be able to improve catatonia associated with schizophrenia [320].

### 5.5. Aducanumab

Aducanumab is an immunoglobulin (IgG1) monoclonal antibody targeting Aß that received FDA approval in 2021. It is a first-of-its-kind AD treatment targeting the fundamental pathophysiology of AD. Aducanumab is approved to treat mild Aß related AD based on clinical trials and targets insoluble Aß fibrils and soluble Aß oligomers in the brain [321,322]. Efficacy of aducanumab was evaluated in three distinct double-blinded, randomized studies with a combined total of 3482 participants. The results of those studies revealed reduced Aß plaques in the brains of treated patients via PET imaging [323]. A drawback of aducanumab is that it can increase amyloid related imaging abnormalities (ARIA), such as brain effusion and hemorrhages, and it is also contraindicated in those with other neurological disorders [207,210].

### 5.6. Lecanemab

Lecanemab is the most recent FDA approved AD treatment. It is an IgG1 monoclonal antibody that also targets Aß [324]. Lecanemab works by targeting soluble aggregated Aß, one of the conformational states of Aß, and binding to it [325]. In mouse models, lecanemab has reduced Aß clusters and plaques, and prevented the build-up of Aß [326]. Based on those results, a phase 2 experiment was conducted, which tested the success of lecanemab compared to placebo in 854 randomized early and mild AD patients. Using a Bayesian design with a response adaptive randomization, this experiment assessed Clinical Dementia Rating-Sum-of-Boxes (CDR-SB), AD Composite Score (ADCOMS), AD Assessment Scale-Cognitive Subscale 14 (ADAS-Cog14), and Mini Mental State Examination (MMSE) every 3 months to monitor drug effectiveness. The study also had two key endpoints: a change in ADCOMS from baseline at 12 months, and a change at 18 months from baseline in ADCOMS, ADAS-Cog14, CDR-SB, total hippocampal volume via volumetric MRI, and optional tests of brain amyloid by PET Standard Uptake Value ratio (SUVr) and CSF biomarkers [327]. The success goal was to have an 80% probability of 25% or higher effectiveness versus the placebo on the ADCOMS. After 12 months, the results were at a 64% probability, better than the placebo by 25%. After 6 more months, lecanemab reduced Aß and was 27% more effective than the placebo on the ADCOMS. This still did not produce the desired results, as 9.9% of patients experienced amyloid abnormalities [327,328]. Longer dosing and longitudinal studies may show greater improvement not observed in the short-term studies. Lecanemab and aducanumab target one component of AD, Aß. While these treatments could be effective for those who have dementia marked by the presence of amyloid and tau aggregation, other therapeutic approaches are needed for other forms of dementia.

### 5.7. Upcoming Treatments

Potential treatments targets that are currently being evaluated fall under three major pathways affected in AD, which are the amyloidogenesis pathway, the tau pathway and the synapse formation pathway (Table 6). Amyloid pathway targets include the enzymes that contribute to formation of toxic Aβ monomers, such as BACE1 and γ-secretase. Inhibitors of γ secretase, like LY411575 and LY-450139, have been tested in both preclinical animal studies and clinical trials [329,330]. Preclinical studies have pointed to the multitude of substrates and the crucial role for γ-secretase for normal cognition and pathways such as Notch signaling, which are likely to have led to some of the observed side effects in clinical trials, such as increased risk of skin cancer and exacerbated cognitive decline [331,332,333]. BACE1 inhibitors, such as MK-8931, AZD3293 and JNJ-54861911, were tested in clinical trials, with phase I/II trials showing the ability to clear CSF Aβ levels; however, results from phase II/III trials were terminated early due to absence of improvement in clinical cognitive symptoms, with JNJ-54861911 also showing liver toxicity [334,335,336]. A lack of efficacy in treating cognitive symptoms also led to termination of the more recent clinical trials for E2609 and CNP520 [337,338]. Late intervention after onset of cognitive symptoms was hypothesized to be one of the reasons for inability of BACE1 inhibitors to treat AD. Early intervention after onset of elevated Aβ levels but before cognitive decline has been proposed as an alternative approach to treatment with BACE1 inhibitors [339]. Some forms of AD do not present with Aβ abnormalities, which necessitate the development of therapies targeting other implicated pathways. Therapies targeting tau currently being studied include the drugs inhibiting NFT formation, LMTM and ACI3024, which have both recently completed phase III clinical trials (NCT03446001, NCT01383161).

Some treatments can also target multiple different pathways, and one potential treatment currently being tested consists of various forms of electromagnetic field (EMF) stimulation. This includes pulsed EMF stimulation, transcranial direct current stimulation (tDCS) and transcranial alternate current stimulation (tACS), which can increase clearance of Aβ and tau aggregates [340,341]. Preclinical studies show that different types of electromagnetic stimulation led to reduced Aβ aggregation in mouse models of AD, with chaperone-mediated degradation and improved mitochondrial function being proposed as possible mechanisms [342,343,344]. Studies in humans showed that weak electromagnetic field stimulation over several weeks resulted in improvement in cognitive function, while strong electromagnetic stimulation led to an increased AD risk [345,346,347].

**Table 6:** Clinical trials and FDA approved treatments for AD. Treatments targeting the 3 major pathways affected in AD: amyloidogenesis, NFT formation and synapse formation. Current and terminated clinical trials along with FDA approved treatments for each category of therapies are described [339,340,348,349]. Clinical trials are ongoing unless the termination date is indicated in parentheses.

Pathway	Intervention	Mechanism	Clinical Trials	FDA Approved Treatments
Amyloid	BACE1 inhibitors: MK-8931, AZD3293, JNJ-54861911, E2609 and CNP520	Inhibits β-secretase, an enzyme that cleaves APP at a site that leads to formation of toxic Aβ monomers	NCT01953601 (2013–2018), NCT02245737 (2014–2018), NCT01978548 (2013–2015), NCT02956486 (2016–2020), NCT03131453 (2017–2020)	None
	γ-secretase inhibitors: LY411575, LY-450139, BMS-708163	Inhibits γ-secretase, which cleaves APP	NCT00594568 (2008–2011), NCT00890890 (2009–2013)	None
	Contraloid Acetate	Disassembly of Aβ oligomers into monomers	NCT04711486, NCT03955380, NCT03944460	None
	Monoclonal antibody: Aducanumab, Lecanemab, LY3372993, Crenezumab	Recognize and bind Aβ or proteins in the Aβ pathway	NCT03720548, NCT03977584	Aducanumab and Lecanemab
Electromagnetic field (EMF) stimulation	low and high frequency pulsed EMF stimulation, transcranial direct current stimulation (tDCS), transcranial alternate current stimulation (tACS)	Clearance of protein aggregates, chaperone-mediated degradation, improved mitochondrial function	NCT02873546, NCT04045990, NCT05784298, NCT01481961	None
NFT pathway	Inhibitors of tau aggregation: LMTM, ACI3024, Curcumin	Inhibits formation of aggregated tau NFTs	NCT03446001, NCT01383161	None
	Tau inhibitors: BIIB080	Inhibits tau protein production	NCT05399888	None
	Antibodies: RG7345, Gosuranemab, Semorinemab, Zagotenemab, JNJ63733657	Recognize and binds tau protein	NCT02281786, NCT03068468, NCT03289143, NCT03518073, NCT04619420	None
Acetylcholine	Cholinesterase inhibitors	Inhibits the enzyme that breaks down acetylcholine	NCT02087865, NCT01951118, NCT02079246, NCT00428389	Donepezil, Rivastigmine and Galantamine

### 5.8. Holistic Treatments

Alternatives to medical intervention are holistic treatments to reduce the modifiable risk factors of AD, such as diet and exercise. Making lifestyle changes has been shown to reduce AD risk in older individuals. Studies have found that exercise can activate brain neurogenesis, plasticity, vascularization, and reduce inflammation by reducing the production of Aß, thus improving cognitive health, and reducing AD [6]. 

The mounting evidence for the gut–brain connection indicates that diet and microbiome are likely to play an important role in AD risk alteration. Bacteria, such as *Lactobacillus* and *Bifidobacterium* species, are thought to play a therapeutic role in AD, and can be acquired through the consumption of foods, like cheese, vegetables, and yogurt [350]. Diets rich in branched-chain amino acids (BCAA) and saturated fatty acids have been shown to promote dementia progression, compared to the well-known Mediterranean diet rich in omega-3 polyunsaturated fatty acids, fiber, and antioxidants, which has demonstrated an AD protective effect [351], likely via the mediating role of SCFAs that can be produced by gut microbiota [352]. A meta-analysis of several studies that measured the association between AD risk and Mediterranean diet adherence revealed a 32% reduced risk with diet adherence [353]. Nutrients and compounds that have shown beneficial effects on AD include caffeine, coenzyme Q10, curcumin, folic acid, glutathione, lecithin, polyphenols, UA, unsaturated fatty acids, and vitamins B6 and B12 [351]. Other diets that have also demonstrated AD prevention effects include the DASH (Dietary Approaches to Stop Hypertension) diet and MIND (Mediterranean-DASH Intervention for Neurodegenerative Delay) diet, the most effective [351].

Sodium oligomannate is a seaweed-derived oligosaccharide drug approved in China for treatment of mild to moderate AD. This approval followed the completion of a phase 3 clinical trial with 818 participants that lasted 36 weeks. The main endpoint for this study was a better change from baseline in the ADAS-cog12 for drug patients compared to placebo. Additional endpoints for this study were better differences in drug to placebo changes for the AD Cooperative Study-Activities of Daily Living (ADCS-ADL) scale, Clinician’s Interview-Based Impression of Change with caregiver input (CIBIC+), and NPI [354]. Sodium oligomannate is proposed to reconstitute the gut microbiome, reduce Aß deposits, and reduce neuroinflammation based on study in animal models [354]. This is yet to be validated in humans.

## 6. Discussion and Conclusions

AD is the leading cause of dementia which is among the top 10 leading causes of death worldwide. On average, once diagnosed with severe AD, patients usually die within 4 to 8 years; however, some have survived up to 20 years following diagnosis [7,355,356,357,358,359,360,361,362,363]. As the medical community works to extend the average human life expectancy beyond 80 years, the prevalence of AD is expected to triple by the year 2050 with the increase in the population of people over 80 years old. This looming crisis adds pressure to the need for methods of earlier diagnosis and treatment options. Recent studies are beginning to show that the brain changes associated with the severe symptoms of AD can begin up to 20 years prior to disease manifestation. Establishing accessible, accurate, and non-invasive tests for CSF and blood biomarkers that detect these early changes is paramount for stemming the occurrence and severity of AD. While biomarkers of AD also overlap with other co-morbid diseases, such as depression and inflammatory diseases, there appears to be specificity in the combination of biomarkers that are differentially expressed in AD and can better inform diagnosis. Given that AD is generally not diagnosed until symptom manifestation in the later stages of life, understanding of the genetic mechanisms of early disease is imperative to earlier diagnosis and treatment, and since identification of people who will develop AD in the future is not currently possible, the next best option would be animal models. Studying the pre-AD changes in animal models could reveal new biomarkers for earlier diagnosis. Further study of biomarkers and imaging tests for early AD could also contribute to methods for early diagnosis. A greater understanding of the major genetic pathways implicated, including the Aβ and NFT pathways, as well as chaperones and Aβ, could also pave the way for novel treatment targets for AD. 

More and more studies show that environment plays a major factor in AD. Connections between microbiome and AD are becoming an important field of research, as it has become more apparent that lifestyle can influence health further down the line. Most treatments for AD are merely symptomatic and do not treat the true degenerative issue. In the past 2 years, two drugs have received FDA accelerated approval to try to meet the need for a therapy that targets one of the main aspects of AD degeneration, which is Aβ plaques. Aducanumab and lecanemab are only approved for mild and early-onset AD and there remains no approved treatment for advanced AD. Studies of the genes associated with AD have made substantial progress in the identification of many susceptibility and protective variants. Future therapies could use these variants to prevent or slow AD progression in patients at every stage of disease. Furthermore, holistic treatments are emerging that focus on modifiable risk factors, such as diet, microbiome and exercise. In conclusion, focus on identification of biomarkers and development of tests for early diagnosis is necessary for more successful treatment of AD. In particular, diagnostic blood biomarkers are important non-invasive methods for predicting and preventing disease. Development of an effective treatment for advanced AD is also an urgent unmet need for current patients who are not eligible for the mild and early-onset treatments currently in the market.

## Figures and Tables

**Table 2:** miRNAs altered in AD patients for potential biomarker diagnostic testing. Certain microRNAs (miRNAs) have been found to have altered levels in AD patients compared to healthy individuals.

Changes in AD Patients	miRNA Biomarkers
Down-regulated	miR-15b-5p, miR-19b-3p, miR-23a, miR-26a, miR-26b, miR-26b-5p, miR-19c-3, miR-31, miR-34a-5p, miR-103, miR-125b, miR-146a, miR-181c, miR-191-5p, miR-193b, miR-222, and Let-7d-5p [17]
Up-regulated	miR-34c, miR-132, miR-181c, miR-206, miR-411, and miR-502-3p [17]

**Table 5:** Animal models of AD. A list of animal models of AD by species, with information on the presence of three important characteristics of AD, including Aβ build-up, formation of tau tangles and neurodegeneration. X refers to the presence of either Aβ build-up, tau or tau-like tangles, or neurodegeneration and synaptic deficits.

Model	Aß Build-Up	Tau or Tau-like Tangles	Neurodegeneration or Synaptic Deficits
**Mice**			
5xFAD	X		X
PDAPP	X		X
Tg2576	X		
APP23	X		X
J20	X		X
TgCRND8	X		
PS2APP	X		
APPswe/PSEN1dE9	X		
Tg-ArcSwe	X		
A7	X		
NL-G-F	X		
rTg4510		X	X
PS19		X	X
3xTg	X	X	X
pR5-183	X	X	
**Rat**			
Tg478/Tg1116	X		
PSAPP	X		
McGill-R-Thy1-APP	X		
TgF344	X	X	
**NHP**			
Aging rhesus macaque	X		X
Aging stump-tail macaque	X		X
Aβ oligomer injection in rhesus macaque	X		X
Aβ and thiorphan injection in rhesus macaques	X		X
**Canine**			
canine cognitive dysfunction (CCD) model	X		X
**Zebra fish**			
*psen1* mutant			
*psen2* mutant			
*app-a* and *app-b* mutants	X		X
**Fruit flies**			
*psn* mutant			X
tau mutant		X	X
Aβ mutant	X		X
Aβ mutation in the eye			
***C. elegans***			
Aβ42 mutation in the muscle	X		X
Aβ mutant + human *APOE ε4* transgene	X		X

## Data Availability

No new data was presented.

## Supplementary Materials

The following supporting information can be downloaded at: https://www.mdpi.com/article/10.3390/bioengineering11010045/s1, Table S1: Variants associated with early-onset Alzheimer’s Disease (EOAD); Figure S1: Genes involved in the Aβ pathway in AD; Figure S2: The tau pathway gene network implicated in AD; Figure S3. APOE and the lipid metabolism pathway are involved in AD; Figure S4: Vascular interaction genes in AD; Figure S5. Chaperones, such as heat shock proteins (HSPs), play important roles in AD pathogenesis; Figure S6. Oxidative stress pathway in AD; Figure S7. Synapse formation gene network; Figure S8. DNA repair genes linked to AD genes, APP and MAPT.
