# Supplementary material for: Alzheimer’s Disease: Models and Molecular Mechanisms Informing Disease and Treatments
Source: Bioengineering (Basel). 2024 Jan 1;11(1):45. doi: 10.3390/bioengineering11010045 (PMC10813760; doi:10.3390/bioengineering11010045)
Supplement: Supplementary file 1 [file bioengineering-11-00045-s001.zip › bioengineering-2746604-supplementary.pdf]

bioengineering

MDPI

Supplementary Tables and Figures

Table S1. Variants associated with early onset Alzheimer's Disease (EOAD). The variants for EOAD are given for the *PSEN1*, *PSEN2* and *APP* genes[1]. All are pathogenic variants unless indicated otherwise.

Gene	Variants
PSEN1	c.236C>T
	c.360A>T
	c.415A>G
	c.424G>A
	c.436A>G
	c.436A>C
	c.438G>A
	c.466_467insTTATAT
	c.488A>G
	c.497T>C
	c.509C>T
	c.520C>A
	c.617G>C
	c.656T>C
	c.698T>C
	c.737C>A
	c.745A>C
	c.749T>C
	c.796G>A
	c.806G>A
	c.811C>G
	c.833G>T
	c.839A>G
	c.839A>C
	c.869-1624_956-2452del
	c.869-2A>T
	c.869-1G>A
	c.869-1G>T
	c.1229G>A
	c.1292C>A
	c.1307C>A
	c.1318_1320del
	g.(?_73673074)_(73673200_?)del
PSEN2	g.(?_226881888)_(226895599_?)del
	c.254C>T
	c.365C>G

	c.422A>T
	c.715A>G
	c.717G>A
APP	c.2149G>T
	c.2149G>A
	c.2143G>A
	c.2141C>T
	c.2140A>G
	c.2080G>A
	c.2078A>G
	c.2077G>C
	c.2075C>G
	c.2018C>T
	c.2010_2011inv
	g.(?_27113910)_(27542938_?)dup
	g.13636378_28138533dup
	A673T (protective)

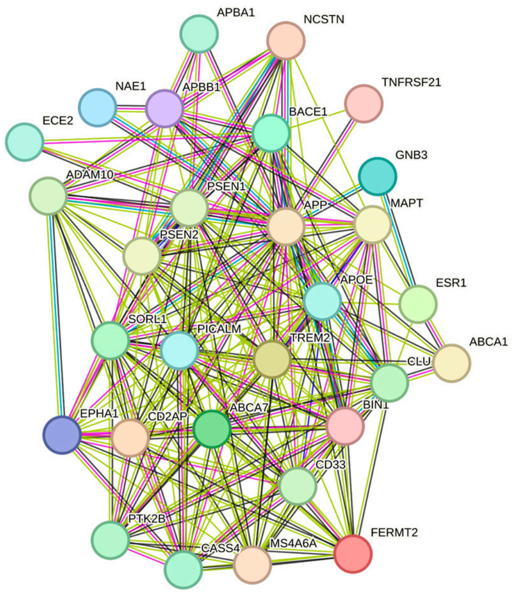

Figure S1. Genes involved in the Aβ pathway in AD. The nodes represent proteins and the edges represent their functional associations (String v10)[2]. Interactions from curated databases are shown in blue and interactions mentioned in publications are given in green, while pink lines depict experimentally determined interactions and green lines represent proteins in close proximity.

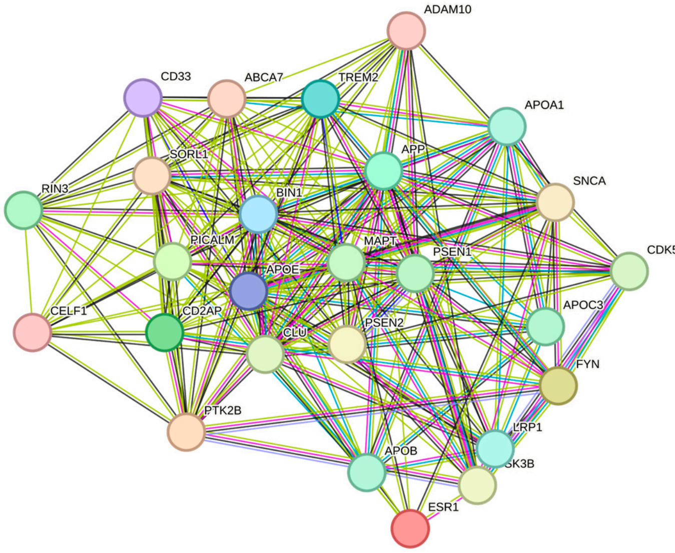

Figure S2. The tau pathway gene network implicated in AD. The nodes depict proteins and the edges show their functional associations (String v10)[2]. Interactions from curated databases are given as blue lines and interactions mentioned in publications are depicted using green lines, while pink lines show experimentally determined interactions and green lines represent proteins in close proximity.

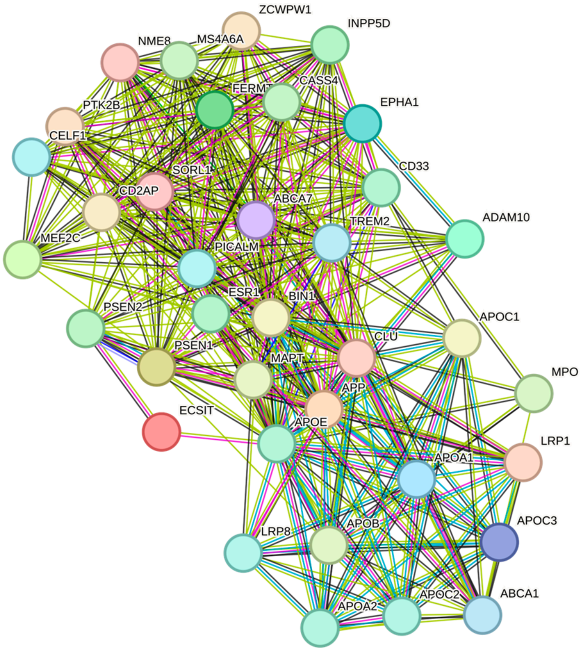

Figure S3. ***APOE*** **and the lipid metabolism pathway are involved in AD.** The nodes show proteins and the lines depict their functional associations (String v10[2]. Interactions from curated databases are given as blue lines and interactions mentioned in publications are depicted using green lines, while pink lines show experimentally determined interactions and green lines represent proteins in close proximity.

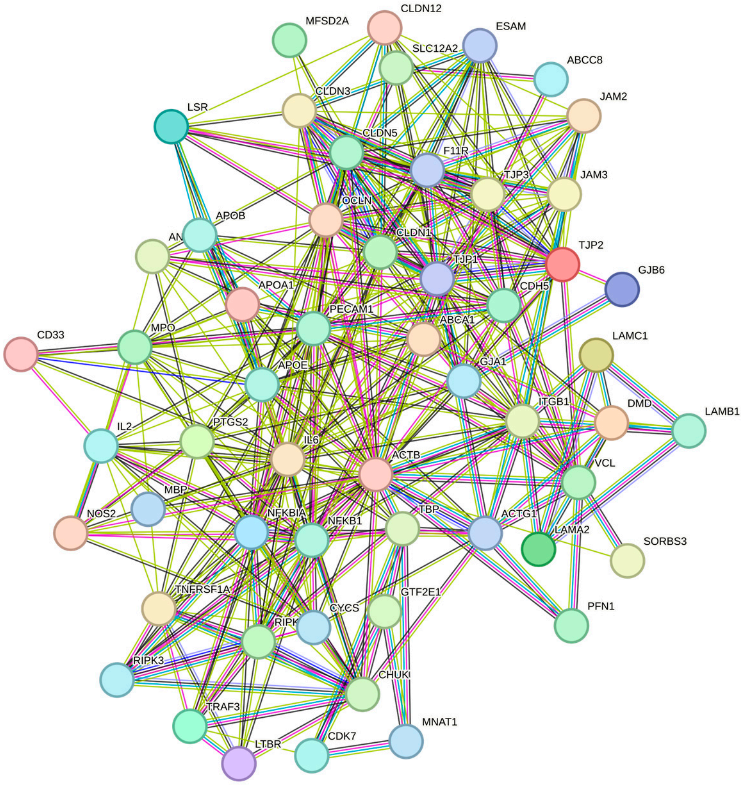

Figure S4. Vascular interaction genes in AD. The nodes show proteins and the lines depict their functional associations (String v10[2]. Interactions from curated databases are given as blue lines and interactions mentioned in publications are depicted using green lines, while pink lines show experimentally determined interactions and green lines represent proteins in close proximity.

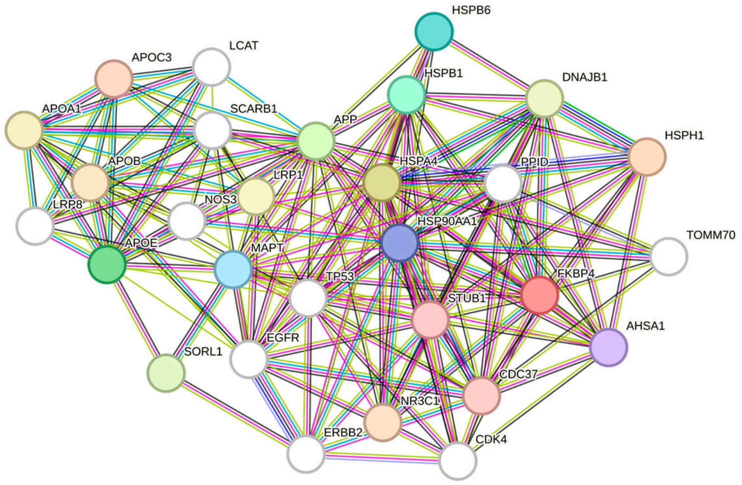

Figure S5. Chaperones, such as heat shock proteins (HSPs), play important roles in AD pathogenesis. The nodes represent proteins and the edges show their functional associations (String v10)[2]. Interactions from curated databases are given as blue lines and interactions mentioned in publications are depicted using green lines, while pink lines show experimentally determined interactions and green lines represent proteins in close proximity.

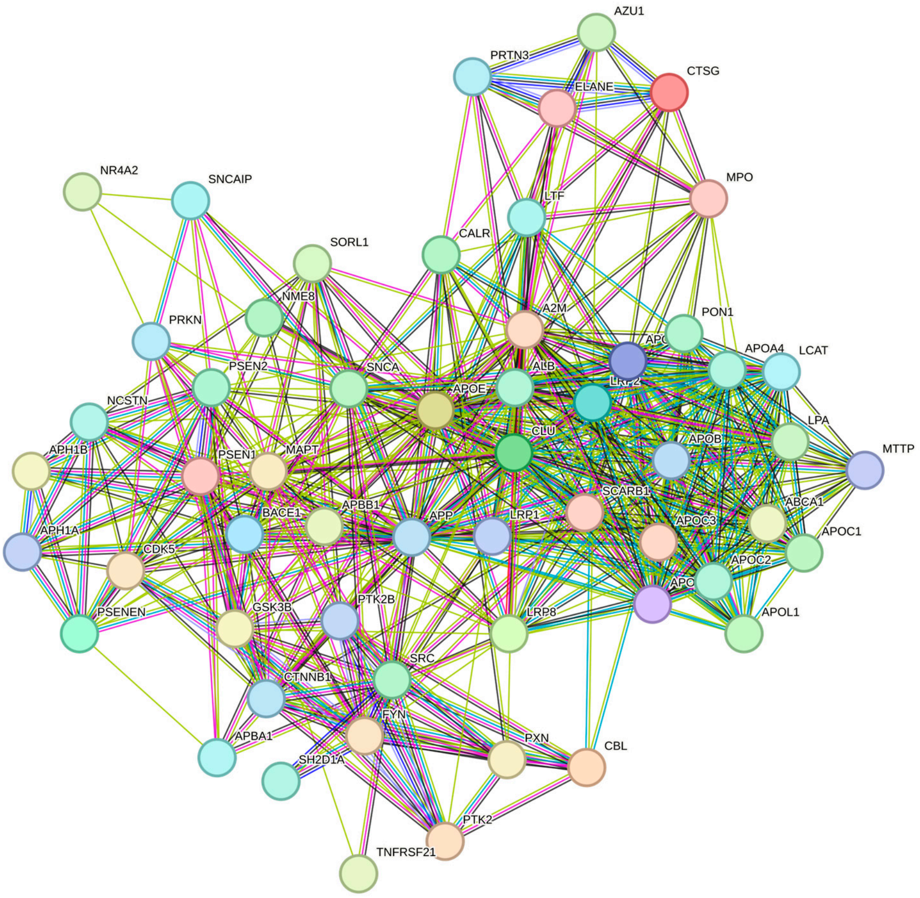

Figure S6. Oxidative stress pathway in AD. The nodes represent proteins and the edges depict their functional associations (String v10)[2]. Interactions from curated databases are given as blue lines and interactions mentioned in publications are shown using green lines, while pink lines show experimentally determined interactions and green lines represent proteins in close proximity.

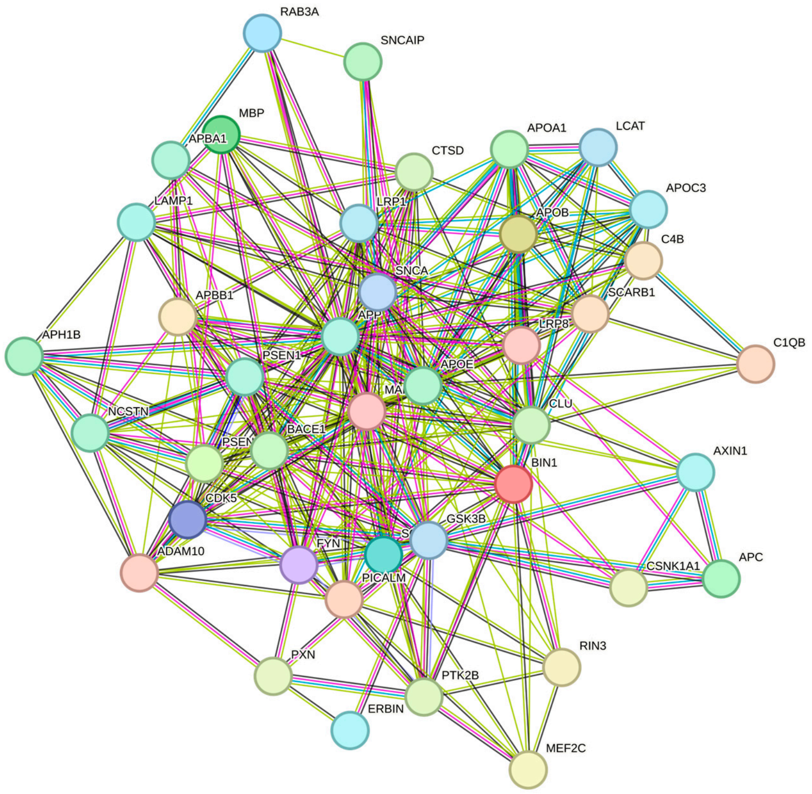

Figure S7. Synapse formation gene network. The nodes represent proteins and the edges represent their functional associations (String v10)[2]. Interactions from curated databases are given as blue lines and interactions mentioned in publications are depicted using green lines, while pink lines show experimentally determined interactions and green lines represent proteins in close proximity.

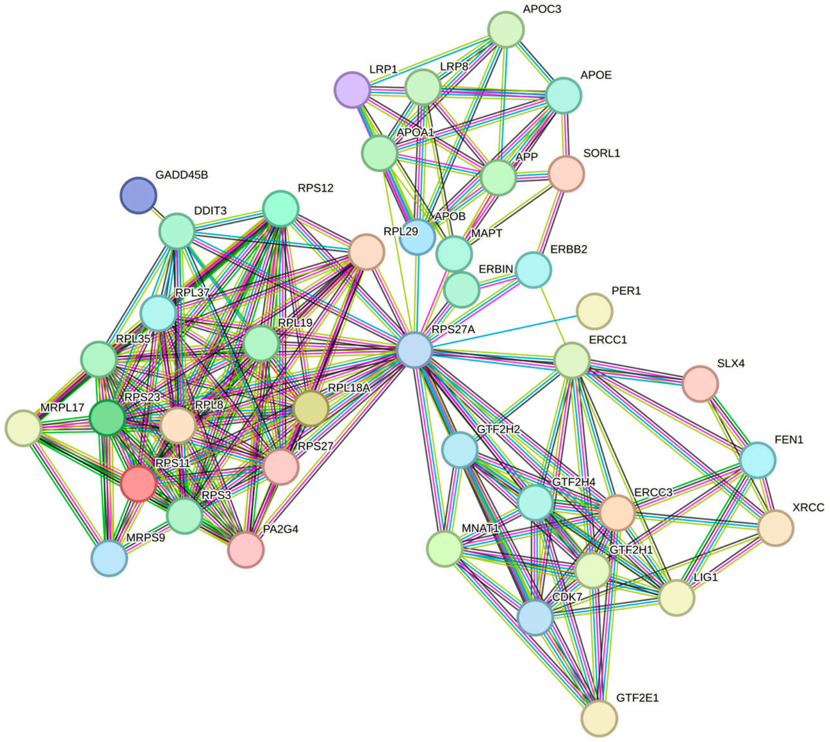

Figure S8. DNA repair genes linked to AD genes, APP and MAPT. The nodes depict proteins and the edges show their functional associations (String v10)[2]. Interactions from curated databases are given as blue lines and interactions mentioned in publications are shown using green lines, while pink lines show experimentally determined interactions and green lines represent proteins in close proximity.

References

1. Landrum, M.J.; Lee, J.M.; Benson, M.; Brown, G.R.; Chao, C.; Chitipiralla, S.; Gu, B.; Hart, J.; Hoffman, D.; Jang, W.; et al. ClinVar: Improving Access to Variant Interpretations and Supporting Evidence. *Nucleic Acids Res* **2018**, *46*, D1062–D1067, doi:10.1093/nar/gkx1153.
2. Szklarczyk, D.; Franceschini, A.; Wyder, S.; Forslund, K.; Heller, D.; Huerta-Cepas, J.; Simonovic, M.; Roth, A.; Santos, A.; Tsafou, K.P.; et al. STRING V10: Protein-Protein Interaction Networks, Integrated over the Tree of Life. *Nucleic Acids Res* **2015**, *43*, D447-452, doi:10.1093/nar/gku1003.
